# The role of friction on skin wetness perception during dynamic interactions between the human index finger pad and materials of varying moisture content

**DOI:** 10.1152/jn.00382.2021

**Published:** 2022-01-19

**Authors:** Charlotte Merrick, Rodrigo Rosati, Davide Filingeri

**Affiliations:** ^1^THERMOSENSELAB, School of Design and Creative Arts, Loughborough University, Loughborough, United Kingdom; ^2^Procter and Gamble Service GmbH, Schwalbach am Taunus, Germany; ^3^THERMOSENSELAB, Skin Health Research Group, School of Health Science, University of Southampton, Southampton, United Kingdom

**Keywords:** friction, hygrosensation, psychophysics, skin, wetness

## Abstract

Mechanosensory inputs arising from dynamic interactions between the skin and moisture, such as when sliding a finger over a wet substrate, contribute to the perception of skin wetness. Yet, the exact relationship between the mechanical properties of a wet substrate, such as friction, and the resulting wetness perception remains to be established under naturalistic haptic interactions. We modeled the relationship between mechanical and thermal properties of substrates varying in moisture levels (0.49 × 10^−4^; 1.10 × 10^−4^; and 2.67 × 10^−4^ mL·mm^−2^), coefficient of friction (0.783, 0.848, 1.033, 0.839, 0.876, and 0.763), and maximum thermal transfer rate (*Q*_max_, ranging from 511 to 1,260 W·m^−2^·K^−1^), and wetness perception arising from the index finger pad’s contact with such substrates. Forty young participants (20M/20F) performed dynamic interactions with 21 different stimuli using their index finger pad at a controlled angle, pressure, and speed. Participants rated their wetness perception using a 100-mm visual analog scale (very dry to very wet). Partial least squares regression analysis indicated that coefficient of friction explained only ∼11% of the variance in wetness perception, whereas *Q*_max_ and moisture content accounted for ∼22% and 18% of the variance, respectively. These parameters shared positive relationships with wetness perception, such that the greater the *Q*_max_, moisture content, and coefficient of friction, the wetter the perception. We found no differences in wetness perception between males and females. Our findings indicate that although the friction of a wet substrate modulates wetness perception, it is still secondary to thermal parameters such as *Q*_max_.

**NEW & NOTEWORTHY** Our skin often interacts with wet materials, yet how their physical properties influence our experience of wetness remains poorly understood. We evaluated wetness perception following naturalistic haptic interactions with materials varying in moisture content, friction, optical profiles, and heat transfer rates. We show that although mechanical parameters can influence wetness perception, their role is secondary to that of thermal factors. These findings expand our understanding of multisensory integration and could guide innovation in healthcare product design.

## Graphical abstract

**Figure:**
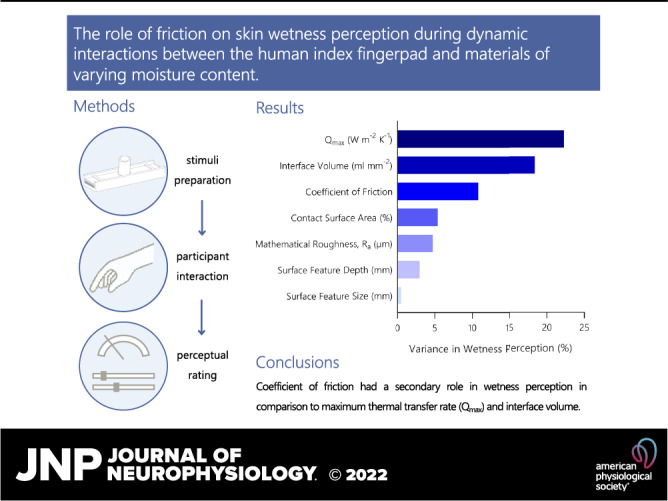

## INTRODUCTION

From birth, humans rely on their hands to provide tactile feedback of their surroundings, from gentle touch to the development of precise manipulation (1). The nature of these interactions is highly variable and complex, with tribological interface behavior depending on innate skin characteristics, material properties, and contact factors such as angle and pressure (2). Another important aspect is interface moisture, as the presence or absence of liquid can significantly affect an interaction, such as by creating lubricating films and altering coefficient of friction (CoF) (3). Consider the impact of wet or sweaty hands when attempting to pick up a glass. Correctly adjusting the required grip force in the presence of moisture is critical to avoid the increased likelihood of slippage, which relies on both tactile feedback and wetness perception (4).

Mechanical parameters, especially CoF, have previously been linked with both roughness perception and wetness perception. Although there is no single physical characteristic that governs the perception of a material, studies report relationships between roughness perception and CoF among several other confounding factors (5–7). Of these, some report CoF to be negatively associated with wetness perception (6), whereas others report a negligible relationship (7). CoF can be defined as the resistance between two surfaces moving against each other and can be calculated as a ratio of tangential to normal force (*Eq. 1*) according to Amontons’ law (8).

Coefficient of friction is calculated as a ratio of tangential force and normal force (8) as follows:

(*1*) $$\text{μ}\;=\;\frac{F_{\text{t}}}{F_{\text{n}}}\;\;\;\;\;\;\;\;\;\;\;\;\;\;\begin{array}{c} \text{μ}=\text{coefficient of friction}\\ F_{\text{t}}=\text{tangential force}\;\left(\text{N}\right)\\ F_{\text{n}}=\text{normal force}\;\left(\text{N}\right) \end{array}.$$

**Figure 1.**
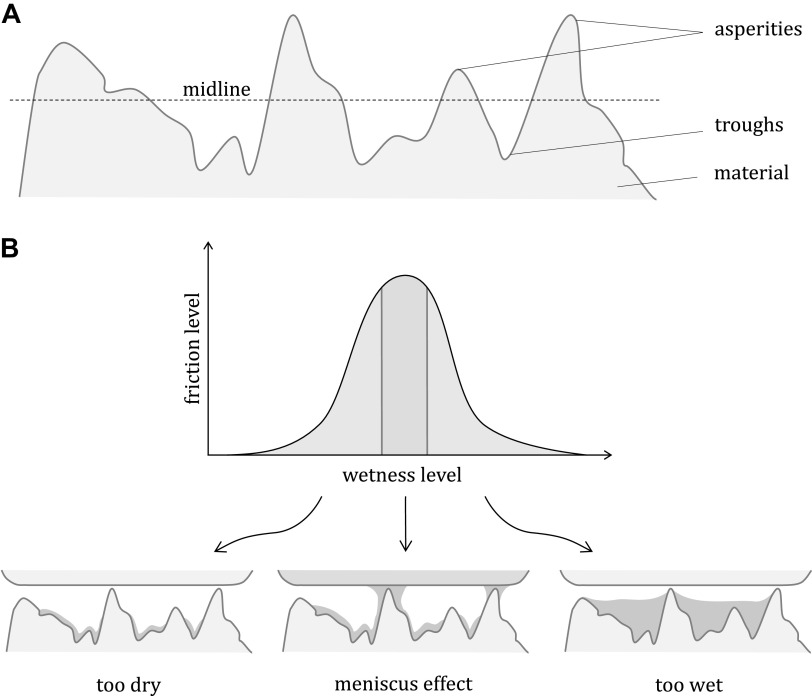
*A*: cross section of material showing the midline with corresponding asperities and troughs that account for the physical topography of the material. *B*: the relationship between interface wetness level and friction level, showing that the capillary bridges causing the meniscus effect can occur at only specific surface tensions associated with different moisture levels.

CoF varies with surface parameters. Another common method of classifying samples is to calculate arithmetic roughness, *R*_a_, in terms of the quantity and magnitude of surface asperities. When observing a cross section of a material, asperities are the peak regions that protrude above the midline and are in contrast to troughs, which fall below the midline (Fig. 1*A*). The relative shape, height, density, and distribution of these asperities within an area are accountable for the material’s frictional properties (9).

Although it may be assumed that a greater magnitude in each of these properties results in greater friction values, this is not always the case (10). For example, P100 sandpaper has a relatively low density of large irregular asperities, which results in a high CoF (11). Contrarily, silicone has a high density of small uniform asperities, but this also results in a high CoF (12, 13). These two materials form a good perceptual contrast, as sandpaper is often considered rough (11), whereas silicone is typically described as smooth (13). However, Childs and Henson (14) found that tactile material differentiation was based more on local CoFs than on overall mean values (2).

Further to this, the interaction between the asperities across two surfaces changes according to the water content of the material, as well as its inherent compressibility, porosity, and absorptivity (15). Consider some drops of water on a table. As you slide your finger across the table, it will initially glide as if lubricated. However, as the water is spread by the finger and the lubricating film begins to break, the finger adopts a juddering motion often referred to as a stick-slip phenomenon (5). In contrast, when both the table and finger are dry again, the movement returns to a smooth glide.

This behavior can likely be attributed to the meniscus effect. This effect occurs at specific water saturations depending on the hydrophilic state, in which capillary bridges form between two substrates to minimize surface tension in the existing water (16) (Fig. 1*B*). Although the presence of capillary bridges is typically associated with adhesion, there is a large variance in the resulting contact mechanics of human skin interactions at different wetness levels that may influence their formation. For example, some studies identify a positive relationship between the presence of capillary bridges and CoF (17), whereas others conclude that their presence is relatively unimportant (16).

The aforementioned principles can be applied to skin tribology. The finger pad is composed of glabrous skin arranged in a series of ridges that collectively form a fingerprint. Although these ridges can effectively be considered as asperities, they do not significantly alter skin surface area between individuals (18). The skin itself behaves as a rubber-like material with viscoelastic properties, such that it has high compliance and can increase contact area with a substrate (19, 20). Although this factor may be a contributor, the CoF at the interface with a substrate also depends on several other properties of the finger. For example, natural variations in skin hydration can be caused by variations in sweat rate (21) or liquid retention in the stratum corneum that acts as a barrier to prevent lower dermal layers from becoming dehydrated and therefore maintains a constant degree of hydration (22). This level of hydration effectively plasticizes the epidermis and may alter CoFs (21).

Further to this, the hydration level of the stimulus itself may affect wetness perception, because of not only physical water content but also the associated thermal conductivity. Wetter samples are typically associated with greater maximum thermal transfer rates, *Q*_max_, such that heat energy transferred from the skin to a lower temperature stimulus will flow at a faster rate (23). As cooling is a main factor in wetness perception (24), it follows that this transfer of heat from the skin would also impact wetness perception.

The nature of the interaction itself is also a significant component of variation in CoF and perceived tactile characteristics. For example, several studies have shown that a greater magnitude of normal force results in a lower CoF (2, 18). However, this result is in contrast to the aforementioned Amontons’ law, which states that the force relationship is independent of surface area and interaction speed under dry conditions and therefore does not support the finding that the magnitude of normal load is inversely proportional to CoF (8). There are additional discrepancies between mechanical and skin research, such as the effect of velocity. Between materials that exhibit viscoelastic properties, as skin does, friction typically decreases at higher velocities (25). However, with skin interactions, there is no consistent relationship with velocity, as CoF is heavily dependent on material and skin conditions (11, 20). In addition, findings suggest that roughness perception is not affected by velocity (26).

The aforementioned perceptions rely on different somatosensory modalities that are transduced and integrated through a range of different sensory pathways. In glabrous skin, such as that found on the finger pad, cold thermal cues are sensed by cutaneous Aδ thermoreceptors. Mechanoreceptors encode different aspects of touch such as stretch, pressure, and vibration and are transduced by Aβ afferents (24, 27, 28). These sensory pathways must be integrated in the brain to form a representative view of an interaction and hence provide a perception of wetness. Cross-afferent integration has also been noted, such as chemical and thermal activation of TRPM8 receptors that can elicit wetness sensations (29, 30). Therefore, there is potential for several differing modalities to be centrally integrated via different neural mechanisms to form an overall perception of wetness.

Although there is extensive existing research surrounding dynamic finger pad interactions with a range of stimuli, these have typically focused on physical measurements such as CoF and *R*_a_, with most psychophysical measures concentrating on perceived roughness but not perceived wetness. Those that do assess perceptual influence typically use solid nonabsorbent and noncompressible materials such as steel, glass, and polypropylene or use these as a backing sheet for thin fabric samples (21, 31). A consistent relationship between perception across substrates has consequently been difficult to determine.

Our aim, therefore, was to model the relationship between mechanical and thermal properties of substrates varying in moisture levels, CoF, and maximum thermal transfer rate (*Q*_max_) and wetness and roughness perception arising from the index finger pad’s contact with such substrates in healthy, young males and females. We hypothesized that wetness perception will be influenced by friction, such that lower CoFs will provide smoother interactions typically associated with higher wetness perception. As a subset analysis, we further hypothesized that because of the greater relative number of skin receptors, females would be more sensitive to wetness than males.

## METHODS

### Participants

A total of 40 participants were recruited to take part in the study, with 20 males (age 22.7 ± 2.7 yr; BMI 23.9 ± 2.5 kg·m^−2^) and 20 females (age 22.4 ± 2.5 yr; BMI 22.0 ± 2.3 kg·m^−2^). This number was established using a sample size calculation with an α value of 0.05, a β value of 0.20, and an effect size (*f*) of 0.785 based on data from pilot studies (G*Power 3.1.9.2 software; Heinrich Heine Universität, Düsseldorf, Germany). Participants were all right-handed, such that the left hand was used to interact with stimuli and the dominant hand was used to complete perceptual scales. Participants were subject to an inclusion criterion, such that all were healthy, nonsmoking individuals between the ages of 18 and 35 yr with a BMI below 30 kg·m^−2^. Individuals were not taking any long-term medication nor did they have any long-term somatosensory disease. All participants had a low alcohol consumption, classified as being below the recommended weekly alcohol intake.

The study design was approved by the Loughborough University Ethics Committee, and the testing procedures were conducted in accordance with the tenets of the Declaration of Helsinki. All participants were informed of the test procedures and given the opportunity to ask questions. All participants completed a health screen questionnaire, and female participants were asked to specify the dates of their most recent menstrual period. All participants gave their informed written consent before participation. Prior to the scheduled testing, participant’s body mass and height were recorded with a scale (ID1 MultiRange, Mettler, Toledo, OH) and stadiometer (HM-250P, Marsden, UK), respectively, to determine their BMI and hence confirm eligibility for the study.

### Experimental Design

The study was conducted as a single-blind psychophysical experiment, such that participants were unaware of information that may bias the results. Diapers were primarily selected as a stimulus for research applications in the context of absorbent product design while also having effective liquid sequestering capabilities, temperature stability, and consistency of liquid spread. In addition, diapers are available in a variety of material types and textures. They are formed of four separate layers, the outermost being an elasticated chassis, above which an absorbent inner core interspersed with superabsorbent polymer granules can absorb and retain high liquid volumes (32). This is covered by an acquisition layer that distributes liquid across the absorbent inner core, and finally a topsheet that is in contact with the skin.

The topsheet can vary in material, for example, cotton or polyester (33), and in design, such as being a plain, netted, or embossed material (34), all of which can contribute to variation in CoF. The superabsorbent capacity depends on the polarity and ionic concentration of the liquid applied (35). This is typically urine in the context of diapers, which can be imitated using a saline solution (36). Use of saline will not compromise perceptual data collection, as wetness sensations are primarily affected by cold thermal inputs (37) and there are minimal dermatological effects of short-term saline exposure (38).

**Figure 2.**
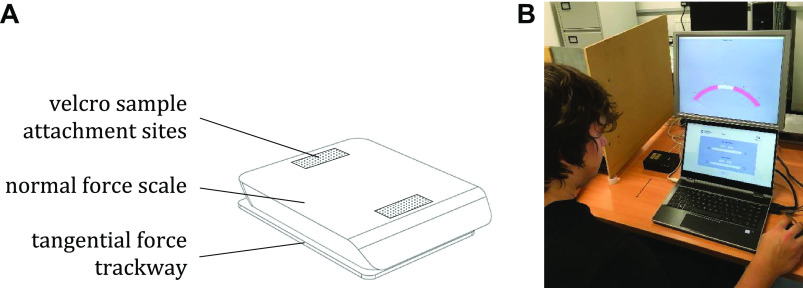
*A*: a diagram of a force plate that measures tangential and normal forces. These measures enabled calculation of coefficients of friction before testing and also allowed in situ coefficient of friction measurements to be recorded for each interaction. *B*: when coupled with an analog meter, the force plate allows real-time normal force feedback to be shown to participants, viewed on the adjacent upper screen. The lower screen shows the visual analog scales used for perceptual ratings.

Each participant attended a single experimental session. Within each session, a series of different stimuli formed of superabsorbent products mounted on a force plate (Fig. 2*A*) with associated analog meter (Fig. 2*B*) were introduced to the participant, who would interact with them by placing their left index finger on the stimulus at a pressure of 2.0 ± 0.5 N and moving their finger toward them at a speed of 50 mm·s^−1^. After this dynamic interaction, but while still in contact with the stimulus, the participant would rate their wetness (very dry to very wet) and roughness perception (very smooth to very rough) of the stimulus, using visual analog scales (Fig. 2*B*).

**Figure 3.**
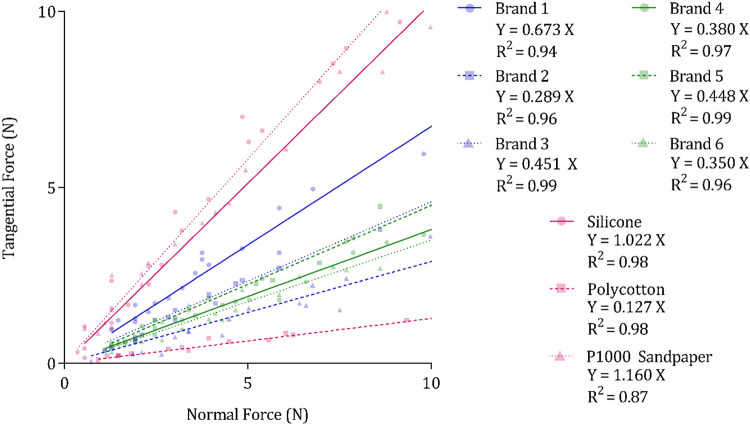
The ratio of tangential force (N) and normal force (N) used to determine coefficient of friction plotted for a range of dry test stimuli and control stimuli, fitted with linear regression. Note that *R*^2^ values are calculated without the forced origin assumed by *Eq. 1*.

Six different diaper brands were used, each characterized by different dry CoFs (0.289 ± 0.047, 0.350 ± 0.040, 0.380 ± 0.039, 0.448 ± 0.032, 0.451 ± 0.029, and 0.673 ± 0.084), which were measured by plotting the tangential forces arising from interactions across a range of normal forces and by calculating the resulting gradient (Fig. 3) (*Eq. 1*). Each diaper was loaded with 40 mL of 25°C saline solution and allowed to rest for a specific time (0 min, 1 min, or 10 min) to achieve one of three different interface volumes (low wetness: 0.49 × 10^−^4 ± 0.20 mL·mm^−2^, medium wetness: 1.1 × 10^−4^ ± 0.07 mL·mm^−2^, and high wetness: 2.67 × 10^−4^ ± 0.22 mL·mm^−2^). This resting period effectively allowed the solution applied to the topsheet to be absorbed by the acquisition layers and wicked away from the application area to ensure uniform distribution. The resulting three interface volumes grouped according to their resting period are herein referred to as mean interface volumes. Wet CoFs measured as a ratio of tangential and normal forces, as mentioned above, across the three different volumes for each diaper brand are referred to as mean CoFs.

This process produced 18 different stimuli varying in wet CoFs (Table 1, *top*). Other characteristics of the stimuli including surface feature size and depth (mm), contact surface area (%), maximum thermal transfer rate, *Q*_max_ (W·m^−2^·K^−1^), and *R*_a_ (µm) varied as a function of the diaper brand used and interface volume. These characteristics were measured using an optical profiler (Primos Compact, GFMesstechnik GmbH, Berlin, Germany) and a textile thermal classifier (Alambeta 1990, Sensora, Liberec, Czech Republic). In addition, we introduced three dry control stimuli including P1000 sandpaper, polycotton fabric, and silicone (Table 1, *bottom*). This addition gave a final total of 21 stimuli (6 diaper brands × 3 interface volumes + 3 controls), which were applied in a balanced order.

**Table 1.** A total of 21 stimuli, including 18 test stimuli formed using 6 diaper brands varying in dry CoF (0.289, 0.350, 0.380, 0.448, 0.451, and 0.673) at 3 interface volumes (low wetness: 0.49 × 10^−4^ mL·mm^−2^, medium wetness: 1.1 × 10^−4^ mL·mm^−2^, and high wetness: 2.67 × 10^−4^ mL·mm^−2^) to form wet coefficients of friction (top) and 3 control stimuli sampled from materials of known physical properties (bottom)

	Test Stimuli																	
Substrate	Brand 1			Brand 2			Brand 3			Brand 4			Brand 5			Brand 6		
Dry CoF	0.289			0.350			0.380			0.448			0.451			0.673		
*R*_a_, µm	38.22			31.64			84.92			197.46			74.4			95.82		
Surface feature size, mm	1.43			—			2.44			1.46			3.24			—		
Surface feature depth, mm	0.37			—			0.63			0.84			1.01			—		
Interface volume, ×10^−4^ mL·mm^−2^	3.47	1.53	0.53	0.76	0.42	0.37	1.51	1.16	0.66	0.14	0.12	0.11	5.77	2.06	0.81	4.38	1.01	0.47
Mean interface volume, ×10^−4^ mL·mm^−2^	1.84			0.52			1.11			0.12			2.88			1.95		
Wet CoF	1.11	1.00	0.87	0.76	1.08	0.72	1.06	1.08	0.96	0.98	0.92	0.74	0.88	0.89	0.82	0.93	0.86	0.49
Mean wet CoF	0.783			0.848			1.033			0.839			0.876			0.763		
*Q*_max_, W·m^−2^·K^−1^)	*	1,128	698	*	973	1,186	*	1,257	892	*	1,206	923	*	741	510	*	1,008	760

	Control Stimuli		
Substrate	Silicone	P1000 Sandpaper	Polycotton
Dry CoF	1.16	1.02	0.13
*R*_a_, µm	*	5.2	4.3
*Q*_max_, W·m^−2^·K^−1^	2,396	491	1,625

CoF, coefficient of friction. Fields marked with a dash (—) indicate that the specified property was not present, while an asterisk (*) indicates values falling beyond the instrument measurement range.

Participants were calibrated and familiarized with protocols before their experimental session. During trials, participants assumed a seated position, and an L-shaped obscuring screen was used to ensure they were blinded to the experimental setup. In addition, auditory cues including stirring and pouring of saline were systematically added every 4 min during stimulus preparation procedures to counteract any associative learning effects or bias in results. This step was preferable to blocking auditory cues entirely, such as using ear defenders, as this would have interfered with the verbal commands used to direct participant interactions. Participants were allowed to take short, self-governed breaks during the trials and were permitted to only consume water during this time. Of the 40 participants, 20 of each sex were recruited to assess sex differences. Within these groups, 10 participants attended morning sessions and 10 attended afternoon sessions as part of a balanced study design.

### Experimental Protocol

Before the start of each experimental session, a 0.9% saline solution was prepared (9.0 ± 0.02 g NaCl; 1,000 ± 10 mL H_2_O). The temperature of the solution was maintained at 25.0°C using a water bath, which is representative of room temperature. A single stimulus was then prepared, varying according to a combination of aforementioned factors, serving as either a test stimulus or control.

The test stimuli were formed from the superabsorbent core, and associated layers were cut from a diaper chassis. Diapers were selected for their high liquid retention abilities, temperature stability, and consistency of liquid spread, as well as for being available in a variety of materials and textures. In each case, 40 mL of saline was applied and the stimulus was allowed to rest for a given dwell time before participant interaction. Control stimuli were also introduced to participants, which consisted of a “positive” reference sample (P1000 sandpaper; rough, high CoF), a “negative” reference sample (polycotton fabric; smooth, low CoF), or a “combination” reference sample (silicone; smooth, high CoF). As these were not superabsorbent stimuli, no saline was applied to them.

The 40 mL of liquid applied to test samples was administered using a specialized acquisition plate (Procter and Gamble GmbH, Frankfurt am Taunus, Germany) formed of a plastic frame and sample stage on to which a plate with an aperture tube would be placed. When the sample was aligned correctly, the tube was positioned directly above the center. The correct volume of solution was then applied via the aperture tube by use of a graduated plastic syringe (SS + 50ES1, Terumo, Leuven, Belgium), which fit firmly within the tube. After the solution had been absorbed from the aperture tube, such that the gauze at the base of the tube was no longer immersed, the sample was removed and allowed to rest for the given dwell time. This period effectively allowed the solution applied to the topsheet to be absorbed by the acquisition layers and be wicked away from the application area, and a consistent liquid spread was maintained within each interaction area.

Before experimental interactions, participants were familiarized with the study protocols by means of demonstrations and they practiced the desired finger positioning, interaction speed, and pressure, as well as any associated commands. Interactions primarily consisted of contacting the stimulus with the left index finger and then dragging it toward them across a 100-mm range at a speed of 50  ± 25 mm·s^−1^ and pressure of 2.0 ± 0.5 N. Pressure was monitored using a specialized force plate (custom made; FS2050 1500 G Load Cell, TE Connectivity, Schaffhausen, Switzerland; LH Series Linear Guides, NSK, Newark, UK; ETH-127-10-13 Peltier Module, Adaptive, Kibworth, UK; M5 Digital Scale, Dymo, Stamford, CT) and continually displayed using an analog meter that could be viewed by the participant during interactions. Conversely, interaction speed was not shown to the participant, as it was more intuitive and could be easily replicated following familiarization. In addition, previous studies have shown minimal effect of interaction speed on roughness perception (39) and as such a wider margin of error was permissible.

After familiarization, participants would undergo a calibration procedure before experimentation to ensure they correctly used psychophysical scales throughout the trial. The calibration protocol consisted of the following four stimuli combinations: rough-wet, smooth-wet, rough-dry, and smooth-dry. Each stimulus was introduced to the participant under standard blinded test conditions, and the corresponding response on the psychophysical assessments was shown. As the stimuli were combinations that demonstrated the extremes of each condition across the experimental sessions, they provided a frame of reference for the study.

Upon completion of the familiarization and calibration procedures, participants inserted their left hand through an aperture in the L-shaped screen. The base of the screen was lined with foam to reduce conductive heat transfer between participants’ skin and the table. When the stimulus had been prepared as aforementioned, the participant was given the command “contact” and they moved their hand downward to make contact. The finger was positioned correctly above the stimulus and moved before contact when necessary.

After contact, participants were prompted to move their finger toward them using the command “move,” by maintaining a speed of 50 ± 25 mm·s^−1^ and a pressure of 2.0 ± 0.5 N. While still in contact with the stimulus to negate evaporative cooling effects, participants completed a digital perceptual form, comprising a 100-mm visual analog scale (very dry to very wet, very smooth to very rough). Participants indicated completion using the word “done,” at which point they would raise their finger and the stimulus would be removed. The participant would then remove their hand from the L-shaped screen and make contact with a custom-made conductance meter using their index finger for 10 s to indicate skin hydration levels and their change between different stimuli and over the course of the session.

A spot measurement of finger temperature was then recorded using an infrared thermometer at a distance of 150 mm (TG56, FLIR Systems, Wilsonville, OR). Subsequently, the participant was instructed “dry,” and they would statically press their index finger for 5 s on to a dry cotton towel to collect residual water. This process was repeated for all stimuli regardless of the wetness level to prevent any learning effect or bias. There was a 4-min period between the presentation of each stimulus, during which time further stimuli were prepared and the aforementioned physiological measures were recorded before repeating the interaction protocol. This time also served as a nervous refractory period.

### Statistical Analysis

In this study, the primary independent variables were interface volume (mL·mm^−2^) and CoF. As different CoFs were accommodated using different types of superabsorbent samples, *Q*_max_ (W·m^−2^·K^−1^) and surface properties including contact surface area (%), surface feature size (mm), surface feature depth (mm), and *R*_a_ (µm) were also incorporated into the analyses. The dependent variables were wetness perception (mm) and roughness perception (mm). Skin conductance (μS), skin temperature (°C), and in situ CoF were also measured as part of methodological validation.

All data were tested for normality of distribution and homogeneity of variances using Shapiro–Wilk and Levene’s tests, respectively. In cases where the assumptions of these tests were violated, parametric means-based tests were nonetheless applied if they best fit the required analyses of the datasets. All statistical data reported in text are mean ($\overset{-}{x}$) ± standard deviation (SD), with means and 95% confidence intervals (95% CIs) given in figures unless otherwise stated; number of participants (*n*) is indicated; α = 0.05. All statistical analyses were conducted using SPSS (Statistical Package for Social Sciences, Version 24.0.0.2, IBM, Chicago, IL). Two-dimensional (2-D) graphical representations of data were produced using GraphPad Prism (GraphPad Prism, Version 8.3.0, GraphPad Software, La Jolla, CA), and three-dimensional (3-D) representations of data were produced using Excel (Excel, Version 2109, Microsoft, Redmond, WA).

The relative contribution of mechanical and thermal parameters, i.e., CoFs, interface volume, *Q*_max_, and of surface properties, including contact surface area (%), surface feature size (mm), surface feature depth (mm), and *R*_a_ (µm), on wetness perception was determined using a partial least squares (PLS) regression analysis. This analysis is used to construct a predictive model when there are many variables that share high collinearity. The method aims to avoid overfitting, in which a model typically fits the data perfectly but that will fail to predict additional data well. The PLS was run with a maximum of seven factors, which is the same as the number of inputted variables. Although similar, variables and factors differ. The term variable refers to the inputted independent variables, which can be grouped within a factor if found to be collinear. Consequently, there will never be more outputted factors than inputted variables.

Each potential number of outputted factors results in the production of a predicted residual sum of squares (PRESS) score, which effectively compares the variance of observed and expected data and predicts how appropriate the model is in terms of its different factors, with the lowest PRESS scores indicating the most suitable models. The analysis was completed using the SIMPLS method with centering and scaling, which accounts for different magnitude ranges and variability in units of data. The leave-one-out cross-validation method was also applied to ensure that the model was internally robust.

A similar PLS assessment was conducted for roughness perception data; however, contributing variables did not exhibit high collinearity, such that the PLS model was not the most applicable to evaluate roughness perception data. Accordingly, the effects of the six mean CoFs on roughness perception were determined by means of linear regression. Similarly, the effects of the three mean interface volumes were determined by means of linear regression. The relationship between magnitudes of wetness perception and roughness perception was assessed using Spearman’s correlation coefficient.

Wetness perception across mean interface volumes was assessed in each sex using a nonparametric Wilcoxon signed-rank test. Similarly, roughness perception across mean CoFs was also assessed in each sex using a nonparametric Wilcoxon signed-rank test.

A one-way repeated-measures ANOVA was used to assess whether change in skin temperature from baseline varied at different mean interface volumes. A one-way repeated-measures ANOVA was also used to assess whether change in skin conductance from baseline varied at different mean interface volumes. These physiological measures were also assessed according to the respective number in the application sequence to determine whether there was a cumulative change in skin conditions over the course of the experiment. The relationship between in situ CoFs recorded for every individual interaction and ex situ mean CoFs was assessed using Spearman’s correlation coefficient to establish interindividual variability.

Finally, wetness perception and roughness perception were compared across the three control stimuli using one-way repeated-measures ANOVA tests with post hoc Tukey tests.

## RESULTS

The assessment of wetness perception in test stimuli using partial least squares regression produced a minimum PRESS score with an output of seven factors. However, Van der Voet scores indicated that this PRESS score did not significantly differ from the second-lowest PRESS score that used an output of two factors. This two-factor output accounted for 65% of variability in wetness perception and involved four of the seven inputted variables [Variable Importance in Projection (VIPs) > 0.8]. This result shows that although the lowest PRESS model will provide a more complete projection of data using seven different factors, it is possible to use a predictive model that still accounts for a large amount of variance with simplified factor contributions. Given the reduced complexity of this model and high response accountability, the two-factor model is best suited and was chosen to describe the data. The PLS regression model generated is presented in *Eq. 2*:

(*2*) $$\begin{array}{c} \text{Wetness perception }\left(\text{mm}\right)=\\ -\text{24}.0\text{9}+\text{26}.\text{45 CoF}\\ +\text{1}0\text{5,1}00\;V+0.0\text{322 }Q_{\text{max}}\\ +0.\text{1414 CS}-0.\text{134}0\text{ SFS}\\ +\text{2}.\text{438 SFD}-0.0\text{277 }R_{\text{a}} \end{array}\begin{array}{c} \text{CoF}=\text{coefficient of friction}\\ V=\text{interface volume }\left({\text{mL mm}}^{-\text{2}}\right)\\ Q_{\text{max}}=\text{maximum thermal transfer rate }({\text{W m}}^{-\text{2}}{\text{K}}^{-\text{1}})\\ \text{CS}=\text{contact surface area }\left(\%\right)\\ \text{SFS}=\text{surface feature size }\left(\text{mm}\right)\\ \text{SFD}=\text{surface feature depth }\left(\text{mm}\right)\\ R_{\text{a}}=\text{arithmetic roughness }(\text{µm}) \end{array}$$

*Equation 2* shows the predictive partial least squares regression equation for wetness perception.

The PLS regression model gives insight into the importance of the respective variables as opposed to the factors (Table 2). It was found that *Q*_max_ accounted for the largest variance in wetness perception across stimuli. This outcome was followed by interface volume and CoF, with other components accounting for less than 10% of variance in wetness perception. There was an unexplained variance of 35.0%.

**Table 2.** Partial least squares regression of wetness perception shown as contributing components and the percentage of variance they account for in the assessed data set

	Variance, %
Maximum thermal transfer rate (*Q*_max_), W·m^−2^·K^−1^	22.3
Interface volume, mL·mm^−2^	18.4
Coefficient of friction	10.8
Contact surface area, %	5.36
Mathematical roughness (*R*_a_), µm	4.73
Surface feature depth, mm	2.97
Surface feature size, mm	0.49
Unexplained variance	35.0

**Figure 4.**
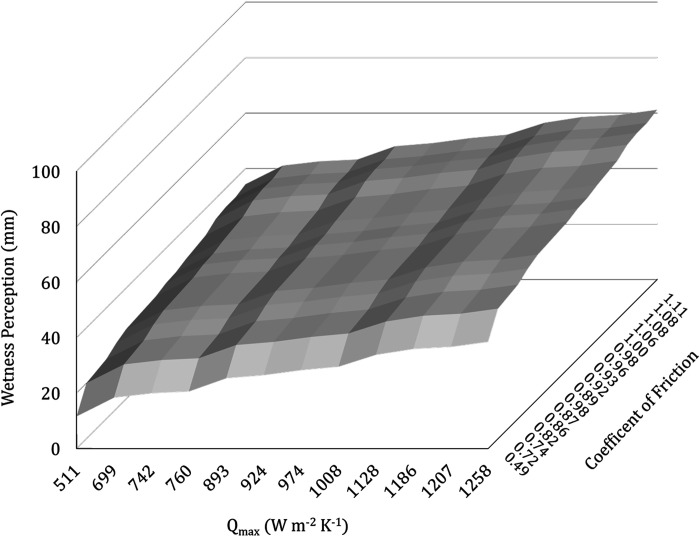
Mean (*x̄*) (*n* = 40) a three-dimensional (3-D) plot showing the effects of maximum thermal transfer rate *Q*_max_ (W·m^−2^·K^−1^) and coefficient of friction on wetness perception (mm). Both maximum thermal transfer rate *Q*_max_ on the *x*-axis and coefficient of friction on the *z*-axis share a positive relationship with wetness perception on the *y*-axis.

**Figure 5.**
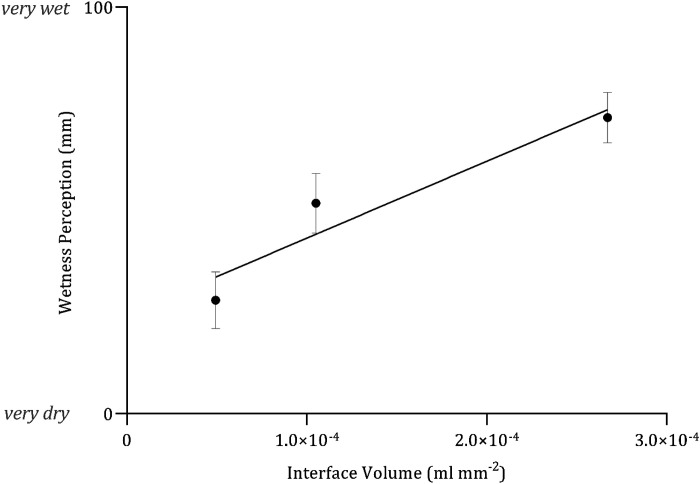
Mean (*x̄*) ± 95% confidence intervals (CIs, *n* = 40) the positive relationship between interface volume (mL·mm^−2^) and wetness perception (mm), with increasing interface volume resulting in heightened wetness perception. Fitted with linear regression, *Y* = 188,960 *X* + 24.30, *R*^2^ = 0.389.

Figure 4 provides a 3-D graphical representation of the relationship between wetness perception, CoF, and *Q*_max_. Figure 5 provides a graphical representation of the relationship between wetness perception and interface volume.

**Figure 6.**
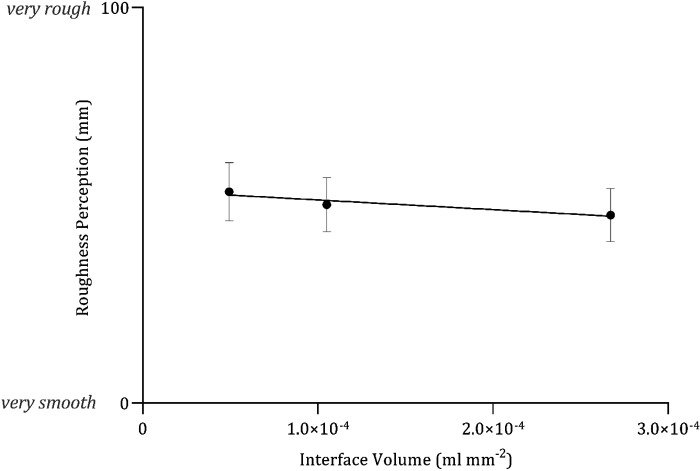
Mean (*x̄*) ± 95% confidence intervals (CIs, *n* = 40) the relationship between interface volume and roughness perception (mm), fitted with linear regression, *Y* = −24,500 *X* + 53.8, *R*^2^ = 0.01.

**Figure 7.**
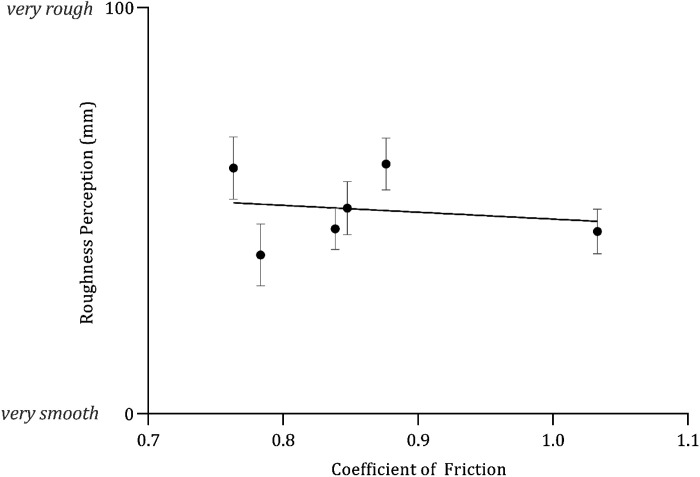
Mean (*x̄*) ± 95% confidence intervals (CIs, *n* = 40) the relationship between coefficient of friction (CoF) and roughness perception (mm). Fitted with linear regression, *Y* = −16.93 *X* + 64.9, *R*^2^ = 0.005.

Roughness perception did not vary as either a function of the mean interface volume (*F*_1,718_ = 2.200, *P* = 0.138, *R*^2^ = 0.01) (Fig. 6) or mean CoF (*F*_1,718 _= 3.358, *P* = 0.0673, *R*^2^ = 0.005) (Fig. 7).

**Figure 8.**
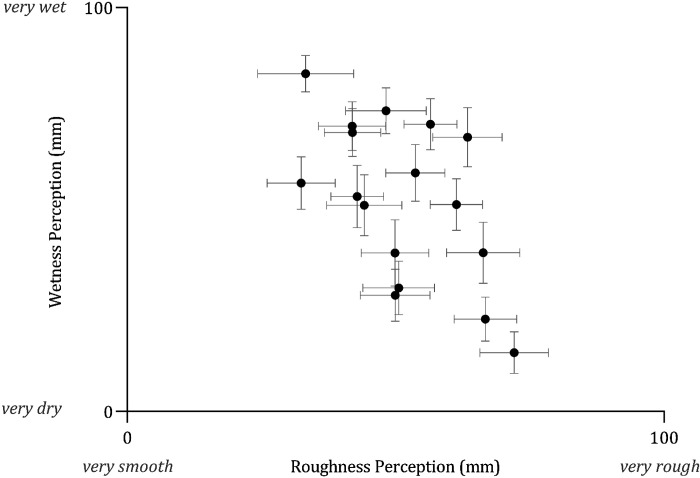
Mean (*x̄*) ± 95% confidence intervals (CIs, *n* = 40) the negative relationship between roughness perception (mm) and wetness perception (mm). Rougher surfaces are perceived as drier and vice versa. Confidence intervals for roughness perception are larger than wetness perception.

Wetness perception was negatively correlated with roughness perception across test stimuli such that substrates perceived as smoother were also perceived as wetter and vice versa [ρ_s_ (718) = −0.183, *P* < 0.001] (Fig. 8).

Males and females perceived wetness similarly across mean interface volumes. Across all interface volumes, the mean wetness perception in males was 51.2 mm, whereas the mean wetness perception in females was 50.5 mm. This deviation is negligible, such that there is no statistically significant effect of sex on wetness perception (*z* = −0.696, *P* = 0.486).

Males and females perceived roughness similarly across mean CoFs. The mean roughness perception in males was 49.7 mm, whereas the mean roughness perception in females was 51.1 mm. This deviation is negligible, such that there was no statistically significant effect of sex on wetness perception (*z* = −0.886, *P* = 0.376).

Change in skin temperature from baseline between interface volumes was not significant (*F*_2,78 _= 0.366, *P* = 0.991) nor did it cumulatively change over the course of each experimental session (*F*_20,780_ = 0.406, *P* = 0.991). In addition, change in skin temperature from baseline did not have a significant effect on wetness perception (*F*_2,78 _= 0.138, *P* = 0.710). Change in skin conductance from baseline did not significantly vary across different interface volumes (*F*_2,78_ = 0.902, *P* = 0.572) nor did it cumulatively change over the course of each session (*F*_20,780 _= 0.214, *P* = 1.000).

**Figure 9.**
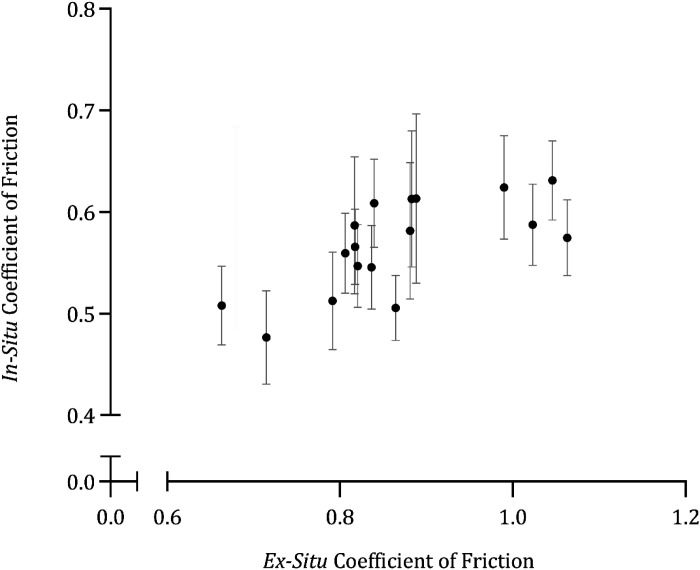
Mean (*x̄*) ± 95% confidence intervals (CIs, *n* = 40) positive correlation between ex situ coefficients of friction and in situ coefficients of friction. Differences in axis magnitudes indicate that in situ coefficients of friction are typically lower than ex situ coefficients of friction.

In situ CoFs shared a positive relationship with ex situ CoFs (Fig. 9) with significant correlation [ρ_s_ (718) = 0.217, *P* < 0.001]. Ex situ CoFs had a significant independent effect on wetness perception (*F*_12,78_ = 37.54, *P* < 0.001), whereas in situ CoFs did not (*F*_5, 195_ = 0.441, *P* = 0.921).

Separate assessment of control samples (Table 3) showed significant variation in wetness perception across materials (*F*_2,78_ = 8.949, *P* < 0.001), with P1000 sandpaper being perceived as significantly drier than both silicone (Δ*x̄* = −14.0; CIs = −5.74, −22.2; *P* < 0.001) and polycotton (Δ*x̄* = −10.9; CIs = −2.64, −19.1; *P* < 0.006). There was also a significant variation in roughness perception (*F*_2,78_ = 221.8, *P* < 0.001), with P1000 sandpaper being perceived as significantly rougher than both silicone (Δ*x̄* = 74.3; CIs = 64.4, 84.2; *P* < 0.001) and polycotton (Δ*x̄* = 77.7; CIs = 67.8, 87.6; *P* < 0.001).

**Table 3.** Mean (x̄) ± SD (n = 40) wetness perception and roughness perception across the three control stimuli of silicone, P1000 sandpaper, and polycotton

	Control Stimuli			
Substrate	Silicone	P1000 Sandpaper	Polycotton
Wetness perception, mm	14.3 ± 20.6	0.30 ± 0.9	11.2 ± 17.3
Roughness perception, mm	12.2 ± 21.3	86.5 ± 20.4	8.80 ± 13.1

## DISCUSSION

The maximum thermal transfer rate, *Q*_max_, had the greatest overall influence on wetness perception and accounted for over a fifth of variance in perception. As the substrate was at a room temperature of ∼25°C, it would have been at a lower temperature than the finger interacting with it, which was ∼33°C. Therefore, heat would have been transferred from the finger to the sample, the rate of which is indicated by *Q*_max_ values. Higher values were associated with greater wetness perception, which effectively indicated that the higher rate of thermal transfer resulting in skin cooling in the finger can be associated with heightened wetness perception. Although the impact of cold thermal inputs has already been noted in wetness perception, the relationship with the thermal transfer properties of the stimulus has not previously been highlighted.

Interface volume was also positively associated with wetness perception. Although the presumed absence of hygroreceptors shows this cannot be a direct relationship, the changes in wetness perception may be related to the influence of *Q*_max_. Higher moisture levels are associated with greater thermal conductivity and therefore a higher maximum thermal transfer rate (40, 41). This outcome may increase the perceived effects of cold thermal inputs such that *Q*_max_ may provide a good indication of wetness perception. In addition, higher interface volumes were likely to trigger more thermal receptors in a given area, further increasing potential wetness perception.

As substrate CoF increased, the wetness perception also increased, forming a positive linear relationship. This outcome was unexpected, given that perceptual research has previously associated higher CoFs with lower wetness perception, such that rough stimuli were perceived as dry and vice versa (6, 42, 43). In terms of roughness perception, neither the interface volume not CoF had a significant effect. This result was unexpected, as roughness perception has long been associated with increases in CoF (44–46), albeit not under wet conditions. The presence of moisture may have affected the dynamics of the interaction by compromising test stimuli fiber structure (47) or by initiating the slip-stick phenomenon as part of the meniscus effect (16).

Although friction and roughness are often associated, CoF is not the only method of measuring friction. For example, the physical roughness parameter *R*_a_ shares a positive relationship with roughness perception. However, *R*_a_ explained less variance compared with CoF in terms of wetness perception, which was the main focus of the study. As wetness perception is better explained by CoF, and roughness perception is better explained by *R*_a_, this study highlights the importance of considering different parameters of the same stimulus.

Differences between roughness perception and physical CoF can also be seen when exclusively observing control stimuli. P1000 sandpaper and silicone have similarly high CoF values of 1.16 and 1.02, respectively, both considerably greater than the CoF value of 0.13 of polycotton, but this difference is not reflected in the roughness perception data. Although P1000 sandpaper has the highest roughness perception values, polycotton and silicone are both considered smooth, highlighting a significant difference between the two materials of similar CoF and again indicating that CoF may not be the most appropriate parameter in describing roughness perception.

Other metrics such as *Q*_max_ may give further insight. This parameter holds its highest value in silicone and the lowest value in sandpaper. A lower *Q*_max_ implies less heat energy is transferred from the finger during an interaction such that the stimulus is comparatively warmer, which has previously been associated with lower wetness perception (48). As this study has identified a negative relationship between wetness perception and roughness perception, it follows that a comparatively warmer stimulus is perceived as rougher. However, this outcome is unlikely to account for the full magnitude of difference in roughness perception between sandpaper and silicone. Therefore, alternative methodologies can be considered to strengthen roughness perception results in both test stimuli and control stimuli.

The aforementioned negative relationship between roughness perception and wetness perception showed that stimuli perceived as rougher than others were generally considered to be drier and vice versa. There are several potential reasons for this, including physical characteristics and learned perceptual associations. As previously mentioned, the properties of a material are typically altered by the addition of moisture, depending on thickness, structure, and pile. Although some of these changes can contribute to increased roughness, there are also a number of changes that result in smoother sensations, which appear dominant here. For example, when the absorbent soft material is wetted, the asperities may lose their structural integrity and become more compressible (47). This compressibility reduces the physical roughness and increases the surface area when in contact with the finger, such that the substrate feels smoother (2). In addition, surface liquid may have contributed to lubricating effects on the surface (3).

Furthermore, smoother surfaces are generally perceived as colder than rough surfaces, as there is greater skin contact area for active heat transfer (49). This cooling may then be further associated with *Q*_max_ and wetness, which is likely in this case, as the moisture applied to the substrate was below the average skin temperature and so would have created a heat sink. There are also learnt and innate aspects that may contribute to the inverse relationship between roughness perception and wetness perception, such as associating smooth wet surfaces with slipperiness. For example, surfaces that are associated with being flat and smooth are also typically considered wet regardless of CoF (50).

The fact that wetness perception and roughness perception shared a negative relationship can be expected and confirms our initial hypothesis. However, wetness perception and roughness perception both share positive relationships with interface volume, implying that as physical wetness increases, both roughness perception and wetness perception will also increase. Instead, they negatively associate with each other, implying another aspect is involved. Although CoF could be this missing link, it results in an insignificant change in roughness perception but an increased response in wetness perception, suggesting each is affected differently.

However, observing CoF in conjunction with interface volume shows that the physical presence of moisture is able to suppress the sensation of roughness, such that roughness perception is not proportional to CoF in wet conditions; there is a dissociation of variables. This outcome implies that the presence of moisture is effectively capable of overriding roughness sensations. It highlights the complexity of the sensory system when presented with multidimensional stimuli that mimic real, tangible interactions, warranting further investigation.

There was no significant difference in wetness perception in males compared with that in females. Although research has previously identified differences in male and female cold perception on the human index finger (51), which is a key driver for wetness perception (24), this parameter did not vary in this study and therefore may account for the absence of sex differences. Some sex differences have also been identified in nonglabrous wetness perception across the body (52), but with varying methodologies that negate robust comparisons.

There was also no significant difference between male and female roughness perception. Although females are associated with having greater receptor density and correspondingly spatial and tactile acuity due to their smaller size (53), they do not typically exhibit a difference in roughness perception compared with males (54), which supports the absence of sex differences in the current research. This outcome could also be attributed to the minimal variation in physical skin CoFs between sexes, which has been established in previous studies (55).

Change in skin temperature from baseline remained consistent across all interface volumes. As skin temperature was recorded after perceptual measurements had been completed and conductance readings were taken, they cannot be directly related to the individual properties of specific stimuli. However, as participants only contacted the stimulus and conductance plate during an interaction cycle, and only the former of these varied in thermal transfer rate and capacity according to interface volume, change in skin temperature can be associated with the said volumes. The fact that the values did not significantly deviate between interface volumes shows that the finger was able to return to a neutral temperature between different interactions.

In addition, as change in skin temperature from baseline remained consistent across the application sequence, there was no cumulative change in temperature across all applied stimuli in a given experimental session. This is important to consider, as temperature has been identified as a key driver in wetness perception (37), and *Q*_max_ has been shown to have a significant influence on wetness perception within specific individual interactions. Change in skin conductance from baseline also remained consistent across all interface volumes. Previous studies have associated skin conductance with skin hydration levels, which in this research could be further associated with the interface volumes of interaction stimuli that may cause such hydration changes (56). Change in skin conductance from baseline also remained consistent across the application sequence, showing that there was no significant cumulative change in skin conductance across stimuli and therefore unlikely to be a change in skin hydration. Although the magnitude of change in conductance and therefore hydration was not significant, the level of hydration required to plasticize the finger and alter CoFs in this context has not yet been determined, and so, the extent to which plasticity may have affected perception is unknown.

Average CoFs recorded in situ for every participant were positively correlated with ex situ CoFs, such that they shared similar relationships with wetness and roughness perception. The ex situ values are likely to be more reliable, as values were determined in a controlled environment with multiple repetitions. The individuals used to establish ex situ CoFs had baseline *T*_sk_ and skin conductance values within 1 standard deviation of the in situ participant means for each. Therefore, the properties of their skin were likely to result in CoF measurements within the natural ranges of the participants.

### Conclusions

We found that CoF and interface volume had a significant effect on wetness perception, with increases in both CoF and interface volume resulting in greater wetness perception. Neither CoF nor interface volume significantly affected roughness perception, but when observed together, showed that the physical presence of moisture is able to suppress the sensation of roughness, such that there is a dissociation of variables. Wetness perception and roughness perception were negatively correlated, such that lower roughnesses were associated with a greater wetness perception. This result highlights an opposing relationship between physical and perceived wetnesses at different roughness levels and shows that there must be an additional or overriding factor in the receipt, transduction, and integration of sensory cues. Neither wetness perception nor roughness perception varied in different sexes. Overall, this outcome gives an insight into the mechanisms underpinning wetness perception and roughness perception using naturalistic interactions and stimuli, emphasizing the importance of tactile cues in their sensory modulation and allowing considerations to be made in both product design and future participant testing.

## GRANTS

The present research was conducted in the context of an industry-co-funded Ph.D. Loughborough University, The Engineering and Physical Sciences Research Council, and Procter and Gamble GmbH provided financial support.

## DISCLOSURES

No conflicts of interest, financial or otherwise, are declared by the authors.

## AUTHOR CONTRIBUTIONS

C.M., R.R., and D.F. conceived and designed research; C.M. performed experiments; C.M. analyzed data; C.M. and D.F. interpreted results of experiments; C.M. prepared figures; C.M. drafted manuscript; C.M., R.R., and D.F. edited and revised manuscript; C.M., R.R., and D.F. approved final version of manuscript.
